# Close cooperation with Health Technology Assessment expertise is crucial for implementation and ultimately reimbursement of innovations in oncology

**DOI:** 10.3332/ecancer.2016.686

**Published:** 2016-10-28

**Authors:** WH van Harten, VP Retèl

**Affiliations:** 1 The Netherlands Cancer Institute, Amsterdam 1066CX, The Netherlands; 2 University of Twente, dept of Healthcare Technology and Services Research, Enschede 7522NB, The Netherlands; 3 Organisation of European Cancer Institutes (OECI), Brussels B 1000, Belgium; 4 Rijnstate Hospital, Arnhem 6800TA, The Netherlands

**Keywords:** Health Technology Assessment, collaboration, innovations, oncology practice

## Abstract

The Organisation of European Cancer Institutes OECI working group on Health Economics and Cost Benefit in Oncology suggests four actions that are needed to improve alignment and integration between clinicians, researchers, and Health Technology Assessment (HTA) experts and agencies: 1) HTA expertise is necessary close to or within the comprehensive cancer centres (CCC); 2) HTA expertise should be physically present throughout the translational research process; 3) Appropriate knowledge is necessary within the research staff; 4) Close cooperation between translational researchers, clinicians, and health economists guarantees clinical ownership.

Fulfilling these conditions may help the translational research field in oncology to interact with agencies and efficiently move innovative technologies through the translational research stages into that of implementation and diffusion. This brings innovative treatments faster to the patient with a greater chance of reimbursement.

## Introduction

The financial sustainability of cancer services is increasingly receiving attention [1]. The OECI has installed a working group on cost benefit/health economics to strengthen this aspect of oncology research throughout Europe and contribute to the development of expertise in this field from the institutional perspective.

At present three parties are the most prominent and active stakeholders in the field of Health Economics: regulators/policy makers, pharmaceutical companies, and HTA experts/research groups.

Regulating agencies such as health insurance (coverage) boards and ministries of health increasingly define health economic criteria for decisions on coverage of innovative treatments. This leads to sometimes complex guidelines and criteria of the file composition which is to be handed over when innovative treatments especially pharmaceuticals are proposed for coverage. Physicians are sometimes involved in advisory boards, but so far they are not very active in the design of the regulations and economic judgement criterias.

Pharmaceutical companies have an obvious interest in positive decisions on coverage requests. They are helped by health coverage-managed care offices with preparation of documentation and verification required in documentation for coverage request files. Although clinical effectiveness and clinical utility are essential elements, these are all marketing oriented as the nature of the coverage decision is decisive for the market potential of drugs. Often this is a bilateral activity between pharma and regulating agencies with limited physician or institutional involvement.

Research groups on health economics are mainly located in universities especially departments of the health services research. For long there has been a tendency to leave this field to those groups and just rely on its outcomes. Strong interaction of HTA-experts with cancer researchers is as of now only realised when they consider clinicians are also essential for coverage decisions, but at that stage it is usually too late to provide clinically relevant input and exert decisive influence on the decision process [2, 3]. In fundamental clinical research groups technology assessment is not considered as contributing factor to their performance, but rather as divertion from the core activities. Partly this is also reflected by their impact. It is noted that the average impact of health services research and pharmacoeconomic journals is presently 1.674 (ISI Web of Knowledge). Gradually we have seen interest from clinicians in that they are adding HTA analyses earlier to their projects whereas fundamental researchers seem to have a larger distance from this field.

In all we can see a division between the most active stakeholders involved in HTA research and in health economic coverage decisions, and the clinicians and institutions that are dependent on those decisions to provide innovative care to their patients.

## Sustainable healthcare and health economic factors

Various parties and papers have drawn attention to the fast growing costs of oncology particularly in the whole healthcare field [1]. Investments needed for diagnostic facilities including imaging such as PET-MRI, genomic analysis and sequencing, robotic surgery techniques, image-guided radiotherapy, and surgery are examples of important cost factors. Especially the high and increasing costs of new drugs and the extensive pipeline of anti-cancer drugs raises concern on the financial sustainability of cancer services. Although the financial crisis may have slowed down the total growth of healthcare costs, almost all underlying technologic and demographic trends point towards further costs increases in the near future. Experts have different opinions on what the maximum ceiling ratio per country related to the gross national product can be, however, it is likely that somewhere in the next decades a maximum (ceiling?) level of collectively supportable healthcare costs will be reached. If a further shift to larger taxation, insurance premiums, and a higher degree of out of pocket costs is to occur, a critical attitude among consumers will result. Both trends will lead to a much higher pressure on care costs and on coverage decisions than those presently conceivable. In combination with the cost developments, this leads to the need for a new balance and new forms of cooperation between the biomedical and especially oncology research field and health economic expertise.

## What is the possible value of closer involvement by the clinical and translational research fields?

So far coverage decisions rely on economic circumstances in lower income European countries and on health economic advice in–especially—western European countries. Examples are the ‘National Institute for Clinical Excellence’ (NICE) in the UK, the ‘Haute Agence de Sante’ (HAS) in France, and the ‘Institut fur Qualitat und Wirtschaftlichkeit im Gesundheitswesen’ (IQWiG) in Germany that generates advice for insurance bodies and governments.

Separately Pharma companies produce files with mandatory information on new drugs or new indications that are filed for reimbursement usually with commercial objectives. In neither of those processes do clinical researchers commonly play an active or leading role, and often their concerns on the outcome of those processes is voiced in a relatively late stage. So far organisations of oncologists—like ASCO and ESMO—try to balance this by producing frameworks that primarily rate drugs through scores on clinical aspects [4, 5]. Some propose alternate models to find a balance between clinical effects and acceptable drug prices [6, 7]. These initiatives are usually only beneficial in late development stages where drugs are entering the market, feeding discussions on price levels, and system sustainability.

A way to exert more influence is to involve HTA expertise earlier in the translational research process and provide input to fundamental and clinical researchers that ultimately makes relevant decision thereby making reimbursement more likely. A second aspect is narrowing the gap and improving mutual understanding between clinicians and HTA experts and regulators. Once they understand each other’s motives and environment, the chance of decision making that is relevant to clinical needs will improve. Obviously government agencies must play their bureaucratic roles, but improving cooperation and providing relevant input might actually contribute to this process and work for the benefit of the patient. It is not a matter of just providing better HTA reports, but it is also securing adequate input for the translational research process to generate better results for society and for regulators to ensure optimal decision making that will encounter more consent from the clinical field and (probably) patient organisations.

**Statement**

The OECI working group on health economics and cost benefit in Oncology suggests that the following actions are needed to improve alignment and integration between clinicians, researchers, and HTA experts and agencies:

1. Health economics- and technology assessment expertise and research capacity should be available within a comprehensive cancer centre or through close cooperation with a health services research faculty.
2. HTA expertise should be physically present in the clinic, near the lab, and in research meetings in order to establish proximity, mutual understanding, and continuous interaction related to innovative research and research designs integrating health economics.
3. Appropriate knowledge should be made available within the research staff. This is needed for one to realise that health economic activities can be of added importance especially in convincing agencies, payers, and governments on the value of research and/or creating the conditions for reimbursement.
4. Close cooperation between translational researchers, clinicians, and health economists guarantees clinical ownership and combined agenda setting in establishing cost effectiveness. This favours speedier translation and actual implementation in clinical practice of innovative technologies.

## Conclusion

Bringing HTA closer to the translational research field in oncology will help select, improve and efficiently move innovative technologies through the translational research stages into that of implementation and diffusion. This will bring innovative treatments faster to the patient with a greater chance of reimbursement.
